# Addison’s Disease Presenting With Idiopathic Intracranial Hypertension in a Young Female

**DOI:** 10.7759/cureus.15195

**Published:** 2021-05-23

**Authors:** Abigail K Fowlie, Muhammad S Majeed, Eleni Karathanasi

**Affiliations:** 1 Endocrinology and Diabetes, Western Sussex Hospitals National Health Service Foundation Trust, Worthing, GBR

**Keywords:** addison's disease, idiopathic intracranial hypertension (iih), headache disorders, primary adrenal insufficiency, hyponatremia

## Abstract

Addison’s disease presenting with idiopathic intracranial hypertension (IIH) is rare but well reported in the literature. IIH has also been reported to occur with other endocrine conditions. We explore some interesting diagnostic and management challenges of a young female that presented with IIH and Addison’s disease. We discuss the features of this unifying neuroendocrine diagnosis.

A previously well 17-year-old female presented to the Emergency Department after a syncopal episode. She had been suffering from worsening and increasing headaches over the last eight months, with vomiting once or twice per day. She had papilledema and reduced visual fields bilaterally. CT head and venogram were normal. Lumbar puncture (LP) opening pressure was raised. She was noted to be hypotensive and hyponatremic. Investigations for hyponatremia revealed random cortisol of <28 nmol/L. She was treated for adrenal crisis. Further investigations were performed and she was diagnosed with IIH associated with Addison’s disease.

Addison’s disease should always be considered in a patient presenting with IIH and hyponatremia. While the mechanism for this association is not completely clear, treating the underlying adrenal insufficiency with steroid replacement alone is an effective treatment and provides symptomatic relief.

## Introduction

A definitive diagnosis of idiopathic intracranial hypertension (IIH) can be made on the basis of raised intracranial pressure and papilledema with normal neuroimaging, cerebrospinal fluid (CSF) composition, and neurological examination (except for cranial nerve abnormalities) [1].

The nomenclature to describe this condition is inconsistent in the literature. Benign IIH has long been discredited due to the potential for irreversible visual impairment. IIH is a diagnosis of exclusion, appropriate for a subset of patients who satisfy the above criteria with no clear secondary cause [1]. There is a significant proportion of patients whereby an underlying etiology for the syndrome is identified. These patients respond to etiology-specific treatments.

IIH has been associated with several endocrine conditions including hyperthyroidism [2], exogenous growth hormone [3], treatment of Cushing’s disease [4,5], secondary adrenal insufficiency [6], and Addison’s disease [7-11]. There have been only seven reported cases of Addison’s disease presenting as IIH, six of which were children [7-11] and one an adult [12].

## Case presentation

Our patient was first seen in the pediatric clinic with a six-month history of frontal headaches. The headaches were occurring every day and were worse in the morning. She reported intermittent visual blurring and morning emesis. Systems examination was normal and funduscopic examination of non-dilated pupils was reported normal. Baseline blood tests were within the normal range. Family history was remarkable for migraines. Sumatriptan was commenced with no effect.

Two months later, she presented with worsening headaches and following a syncopal episode. The headaches were now associated with vomiting multiple times a day. She described a ‘fog’ over her eyes with intermittent double vision and bilateral tinnitus. She reported some recent weight loss and felt light-headed on standing. She had no other systemic symptoms, there were no growth concerns and she had a regular menstrual cycle. There was no past medical history. She was on no medication or contraceptive pill at the time of admission.

**Figure 1:**
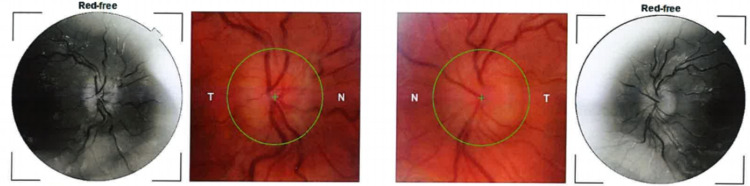
Retinal photographs of our patient showing papilledema.

On examination, she appeared slim with a BMI of 20 kg/m2. She had blood pressure (BP) of 89/48 mmHg and a pulse of 91 beats per minute. The fundal examination was remarkable for bilateral disc swelling (Figure 1), which was further confirmed on optical coherence tomography scanning. Humphrey visual field testing showed reduced visual fields, on the right more so than the left. Neurological examination was otherwise normal.

Laboratory blood tests disclosed isolated hyponatremia of 126 mmol/L. CT head and CT venogram showed no abnormalities. Lumbar puncture (LP) performed in the lateral decubitus position revealed raised opening pressure of 36 cm H20, reduced to a closing pressure of 12 cm H20 after therapeutic drainage. CSF had a normal composition and was sterile.

Following LP, her headaches improved and she was commenced on acetazolamide 250 mg twice daily for presumed IIH. However, just one day later the vomiting returned and the clinical picture appeared unchanged. Subsequent MRI disclosed no abnormalities. Repeat blood tests showed persistent hyponatremia, which prompted further investigations.

**Figure 2:**
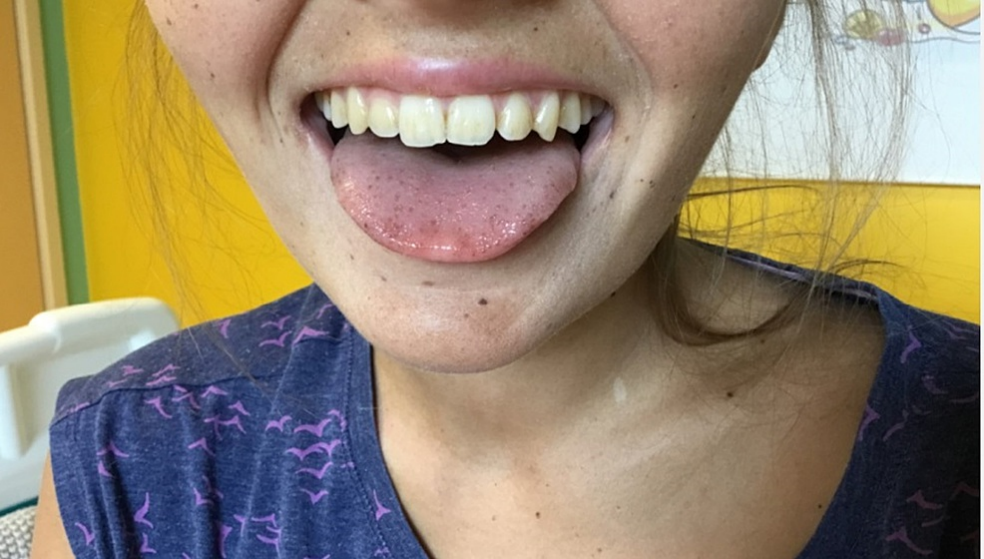
A picture showing the pigmentation of our patient's skin and tongue.

Random serum cortisol was measured and found to be extremely low (<28 nmol/l). She was commenced on intravenous hydrocortisone and 0.9% saline infusion. It was at this point that the diffuse hyperpigmentation of her skin (incongruent to her mother’s complexion) was recognized (Figure 2). This had not been documented previously and we suspect this was because she was always examined in a darkened room. On discussion with the patient and her mother, they assumed she had retained her "tan" well since their summer holiday last year.

A comprehensive set of endocrine and autoimmune laboratory blood tests was performed (Table 1). Abnormal results included low serum aldosterone (68 pmol/L) and increased plasma renin activity (894 mu/L). Unexpectedly, morning plasma adrenocorticotropic hormone (ACTH) was low at 9 ng/L. She underwent adrenal stimulation with a 250 mcg short synacthen test. Basal cortisol level was 17 nmol/L. Subsequent serum cortisol concentrations were 17 nmol/L and 20 nmol/L at 30 and 60 minutes, respectively. High titre adrenal antibodies were detected. Despite the initial low plasma ACTH, the clinical and biochemical picture confirmed Addison’s disease in this patient. The hyponatremia corrected with corticosteroid replacement. Repeat ACTH level at a later date revealed a high ACTH level of 3259 ng/L.

**Table 1:** Table displaying the results of blood tests performed.

Blood test	Result
Sodium	126 mmol/L
Potassium	4.3 mmol/L
Urea	5.6 mmol/L
Creatinine	55 ⴗmol/L
Estimated Glomerular Filtration Rate	>90 ml/min
Serum Osmolality	273 mmol/kg
Urine Osmolality	254 nmol/L
Urine Sodium	24 nmol/kg
Adrenocorticotropic Hormone (ACTH)	Initially 9 ng/L, later 3259 ng/L
Aldosterone	68 pmol/L
Renin	894.3 mu/L
Aldosterone:Renin Ratio	0
Adrenal Antibodies	Positive
Baseline Cortisol	17 nmol/L
Cortisol 30 Mins After Tetracosactide	17 nmol/L
Cortisol 60 Mins After Tetracosactide	20 nmol/L
Thyroid-Stimulating Hormone	0.56 mu/L
Thyroxine (T4)	13.5 pmol/L
Thyroid Peroxidase Antibodies	2.2 iu/L
Insulin-like Growth Factor-1	32.9 nmol/L
Prolactin	468 mu/L
Luteinizing Hormone	4.1 iu/L
Oestradiol	308 pmol/L
IgA Tissue Transglutaminase Antibodies	0.3 U/ml
Liver Kidney Microsomal Antibodies	Negative
Smooth Muscle Antibodies	Negative
Mitochondrial Antibodies	Negative
Gastric Parietal Cell Antibodies	Negative
Anti-Nuclear Antibodies	Negative
Intrinsic Factor	<0.5 u/mL

She was discharged on acetazolamide, hydrocortisone, and fludrocortisone. She was reviewed over the proceeding months with difficulty tapering the dose of hydrocortisone, due to persisting tiredness and occasional vomiting even on higher doses of hydrocortisone. On one occasion her sodium was also noted to be low at 130 mmol/L despite high doses of oral hydrocortisone. Acetazolamide was discontinued and her symptoms improved with no regression in her ophthalmology examination.

## Discussion

The mechanisms for the association between IIH and Addison’s disease are not certain. It has been postulated that IIH arises from an increase in CSF volume secondary to delayed CSF absorption [13]. Experimental studies have shown delayed CSF absorption, without ventricular dilatation due to increased resistance of flow across absorptive channels following acute steroid withdrawal [13]. The putative effects of acute corticosteroid withdrawal on CSF secretion, implicate the enzyme 11β-hydroxysteroid dehydrogenase type 1, an enzyme highly expressed in choroid plexus epithelium that converts inactive cortisone to active cortisol [14]. Cortisol can activate the mineralocorticoid receptors in the choroid plexus with similar affinity to aldosterone, leading to active sodium secretion by the Na/K ATPase at the choroid plexus membrane, movement of sodium ions into the cerebral ventricle, and an osmotic gradient to drive CSF secretion [14].

Our case signifies diagnostic and management challenges to clinicians. Both Addison’s disease and IIH presented at the same time with an initial focus on a presumed diagnosis of IIH. Serum sodium which was low on admission was missed initially and later baseline workup for hyponatremia revealed extremely low cortisol levels. Raised renin levels with low aldosterone supported the diagnosis of primary adrenal insufficiency, however, ACTH levels were falsely low with no clear explanation. ACTH analysis was done by Siemens IMMULITE assay and the initial low level was related to possible mishandling/degradation or spurious analysis.

In the proceeding months after discharge, we postulated that there was impaired absorption of hydrocortisone due to difficulty in tapering the dose. The malabsorption screen was negative and adjusting the timing of her medication had no effect. In the British National Formulary (BNF) [15], acetazolamide and hydrocortisone are not recorded to interact. However, in this case, we have had difficulty managing adrenal insufficiency with the concomitant use of acetazolamide. This may be because some of the uncommon documented side effects of acetazolamide mimic those of adrenal insufficiency; namely vomiting and tiredness [15]. On discontinuation of acetazolamide, she was able to reduce the dose of hydrocortisone and was discharged from ophthalmology follow-up. Comparing other case reports, the majority of IIH associated with Addison’s disease responded well to steroid replacement alone, and only two authors combined this for a short period with acetazolamide [8, 12].

## Conclusions

This case highlights the importance of identifying secondary causes in patients that present with IIH. While IIH is an unusual presentation of the Addisonian crisis, the clinician should be aware of this association, and Addison’s disease should always be considered when hyponatremia co-exists with IIH. Although acetazolamide is the standard treatment for IIH alone, early steroid replacement in Addison’s associated with IIH controls symptoms and may reduce the risk of visual loss.
